# Publisher Correction: Biporous silica nanostructure-induced nanovortex in microfluidics for nucleic acid enrichment, isolation, and PCR-free detection

**DOI:** 10.1038/s41467-024-46401-w

**Published:** 2024-03-05

**Authors:** Eunyoung Jeon, Bonhan Koo, Suyeon Kim, Jieun Kim, Yeonuk Yu, Hyowon Jang, Minju Lee, Sung-Han Kim, Taejoon Kang, Sang Kyung Kim, Rhokyun Kwak, Yong Shin, Joonseok Lee

**Affiliations:** 1 Department of Chemistry, Hanyang University, Seoul, 04763 Republic of Korea; 2 Research Institute for Natural Science, Hanyang University, Seoul, 04763 Republic of Korea; 3 Research Institute for Convergence of Basic Sciences, Hanyang University, Seoul, 04763 Republic of Korea; 4 Department of Biotechnology, College of Life Science and Biotechnology, Yonsei University, Seoul, 03722 Republic of Korea; 5 Department of Mechanical Convergence Engineering, Hanyang University, Seoul, 04763 Republic of Korea; 6 Bionanotechnology Research Center, Korea Research Institute of Bioscience and Biotechnology (KRIBB), Daejeon, 34141 Republic of Korea; 7 Department of Infectious Diseases, Asan Medical Center, University of Ulsan College of Medicine, Seoul, 05505 Republic of Korea; 8 Center for Augmented Safety Systems with Intelligence, Sensing and Tracking (ASSIST), Korea Institute of Science and Technology (KIST), Seoul, 02792 Republic of Korea

**Keywords:** Assay systems, Microfluidics, Mechanical engineering, Structural properties, Biosensors

Correction to: *Nature Communications* 10.1038/s41467-024-45467-w, published online 14 February 2024

The original version of this Article contained an error in the introduction, which incorrectly read ‘However, during the asymptomatic or pre-symptomatic period, the low concentration of the pathogen poses challenges to detect without sample preparation, such as pathogen a’. The correct version adds ‘nd nucleic acid enrichment/isolation.’ after ‘such as pathogen a’. This has been corrected in both the PDF and HTML versions of the Article.

