# Supplementary material for: Biporous silica nanostructure-induced nanovortex in microfluidics for nucleic acid enrichment, isolation, and PCR-free detection
Source: Nat Commun. 2024 Feb 14;15:1366. doi: 10.1038/s41467-024-45467-w (PMC10866868; doi:10.1038/s41467-024-45467-w)
Supplement: Supplementary file 1 — Supplementary Information [file 41467_2024_45467_MOESM1_ESM.docx]

**Supplementary Information**

**Biporous silica nanostructure-induced nanovortex in microfluidics for nucleic acid enrichment, isolation, and PCR-free detection**

Eunyoung Jeon^1,2,3,9^, Bonhan Koo^4,9^, Suyeon Kim^1,2,3,9^, Jieun Kim^5^, Yeonuk Yu^5^, Hyowon Jang^6^, Minju Lee^4^, Sung-Han Kim^7^, Taejoon Kang^6^, Sang Kyung Kim^8^, Rhokyun Kwak^5^*, Yong Shin^4^*, Joonseok Lee^1,2,3^*

^1^Department of Chemistry, Hanyang University, Seoul 04763, Republic of Korea

^2^Research Institute for Natural Science, Hanyang University, Seoul 04763, Republic of Korea

^3^Research Institute for Convergence of Basic Sciences, Hanyang University, Seoul 04763, Republic of Korea

^4^Department of Biotechnology, College of Life Science and Biotechnology, Yonsei University, Seoul 03722, Republic of Korea

^5^Department of Mechanical Convergence Engineering, Hanyang University, Seoul 04763, Republic of Korea

^6^Bionanotechnology Research Center, Korea Research Institute of Bioscience and Biotechnology (KRIBB), Daejeon 34141, Republic of Korea

^7^Department of Infectious Diseases, Asan Medical Center, University of Ulsan College of Medicine, Seoul 05505, Republic of Korea

^8^Center for Augmented Safety Systems with Intelligence, Sensing and Tracking (ASSIST), Korea Institute of Science and Technology (KIST), Seoul 02792, Republic of Korea

^9^These authors contributed equally: Eunyoung Jeon, Bonhan Koo, Suyeon Kim.

* e-mail: [joonseoklee@hanyang.ac.kr](mailto:joonseoklee@hanyang.ac.kr); [shinyongno1@yonsei.ac.kr](mailto:shinyongno1@yonsei.ac.kr); [rhokyun@hanyang.ac.kr](mailto:rhokyun@hanyang.ac.kr)

**Note S1**

**Characterization of LnNPs modified with DNA oligo**

We synthesized inert-core/active-shell/inert-shell LnNPs (NaYF_4_@NaYF_4_, Yb_48_, Tm_2_@NaYF_4_) through a thermal decomposition method and employed it as an LRET donor. The average diameters of monodispersed core, core/shell, and core/shell/shell LnNPs were 12.8±0.5 nm, 21.8±0.8 nm, and 24.2±0.9 nm, respectively, evidenced by transmission electron microscopy (TEM) images (Supplementary Fig. 14a). Energy dispersive X-ray spectroscopy (EDS) mapping images indicated that the core/shell/shell nanoparticles were mainly composed of Na, Y, and F as a lattice host, with increased distribution of the dopant ions (Yb^3+^, Tm^3+^) in the outer region of LnNPs (Supplementary Fig. 14b). An X-ray diffraction (XRD) analysis (Supplementary Fig. 14c) showed that the LnNPs consisted of hexagonal NaYF_4_ (JCPDS:16-0334), which is an effective upconverting host lattice structure owing to its low lattice phonon energy ^1,2,3,4^. As shown in Supplementary Fig. 14d, emission intensity at 800 nm of LnNPs was approximately 79-fold enhanced by coating an inert-shell.

We conjugated the LRET donors with the capture DNA oligonucleotide complementary to a target site of SARS-CoV-2 RNA (Supplementary Fig. 15a). The hydrophobic surface of the an-synthesized donors led us to perform ligand exchange into hydrophilic amine functionalities prior to DNA conjugation. The amine-modified donors (NH_2_-donors) were sulfosuccinimidyl 4-[N-maleimidomethyl] cyclohexane-1-carboxylate (sulfo-SMCC)-treated, and sequentially bound to the thiolate DNA via thiol-maleimide reaction^5,6^. The Fourier transform infrared (FT-IR) peaks for each ligand exchange step from the as-synthesized donors to DNA-modified donors (denoted as DNA-donors) indicated the successful chemical conjugation of DNA oligo onto the donors (Supplementary Fig. 15b). For the as-synthesized donors, transmission bands for the COO- (1449 cm^-1^) and CH_2_ groups (2850 and 2923 cm^-1^) were observed, which were attributed to the presence of oleic acid on the surface. Two new bands for the NH_2_ group at 1643 and 3245 cm^-1^ were observed after surface modification with amine functionalities ^7^. The resulting DNA-donors showed two bands for C-O groups of ribose (1057 cm^-1^) and NH_2_ group (1673 cm^-1^), which were attributed to DNA ^8^. We further confirmed the successful immobilization of DNA using dynamic light scattering (DLS) and zeta potential measurements. The hydrodynamic diameter of the donors gradually increased after the sulfo-SMCC treatment and DNA conjugation (Supplementary Fig. 15c). The surface charge of +33.8 mV for NH_2_-donors decreased to -32.7 mV after sulfo-SMCC treatment, which eventually increased to -28.3 mV after the immobilization of DNA oligo (Supplementary Fig. 15d).

**Core/shell/shell structure for effective LRET**

High-sensitive LRET-based detection of target RNA is achieved by improving LRET efficiency. LRET is a process of short-range resonance energy transfer (RET), which occurs under conditions of the spectral overlap of the donor's emission and the acceptor's absorption, and donor-acceptor spatial proximity^9,10,11^. Nanoparticle-based LRET technologies using active-core nanoparticles show limitations including their dimensional problem such as the limited distance from the donor to the acceptor. We employed an approach to construct an inert-core/active-shell/inert-shell LnNP as an LRET donor for effective LRET (Supplementary Fig. 14). The NaYF_4_ inert-core was encapsulated in a NaYF_4_, Yb, Tm active-shell, which exhibits a strong emission peak at 800 nm under 980 nm excitation. Spatial confinement of an activator (Tm^3+^) within the active shell shortened the energy-transfer distance, increasing the number of activator ions participating in effective energy transfer^7,12,13^. Finally, the inert-core/active-shell nanoparticles were encapsulated in a NaYF_4_ inert-shell to minimize surface defect-induced energy quenching and increase upconversion photoluminescence intensity at 800 nm. The outermost inert-shell was synthesized with ultra-thin thickness (~ 1.2 nm) to achieve an efficient LRET process (Supplementary Fig. 14a).


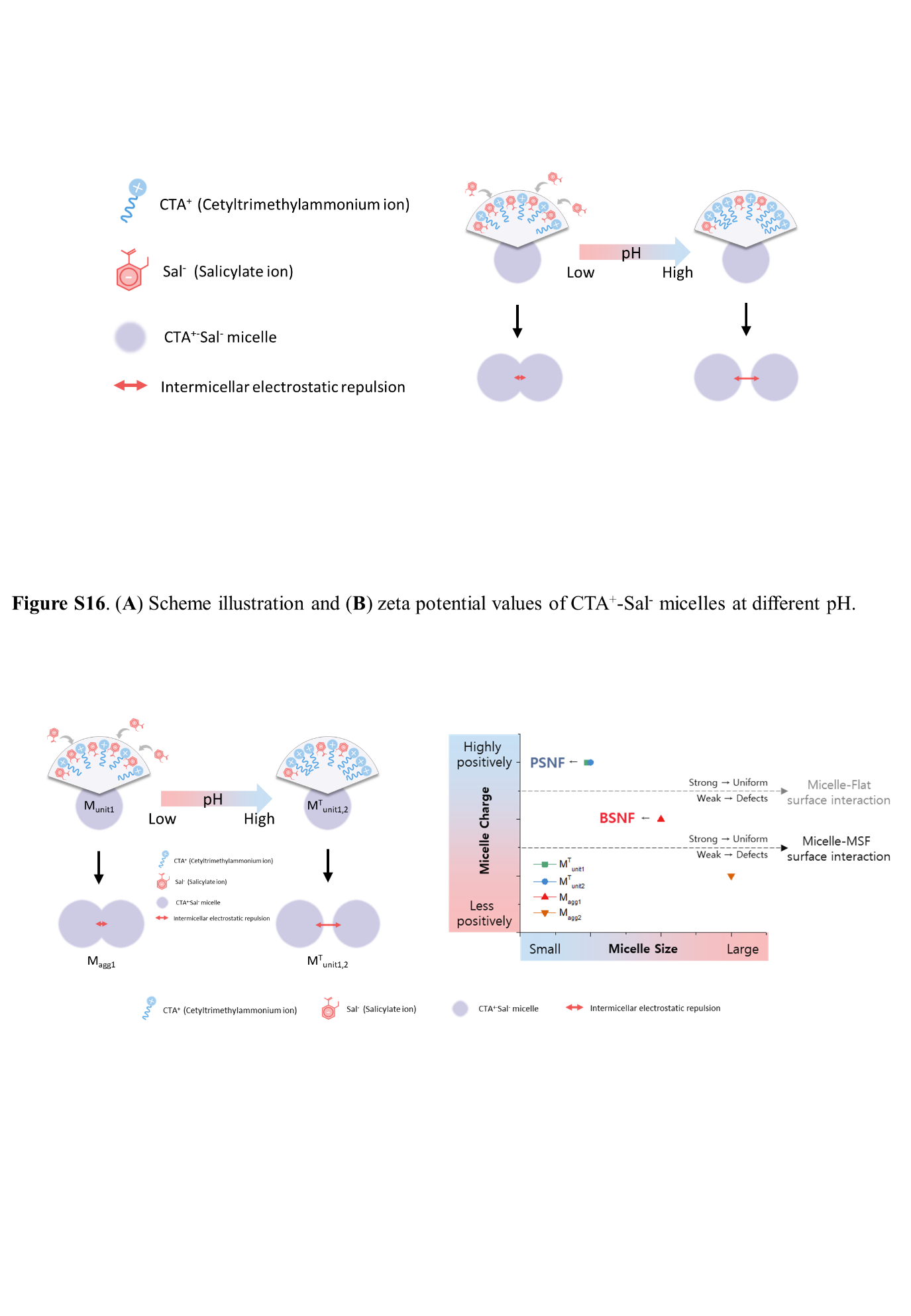


**Supplementary Fig. 1.** **Scheme illustration of CTA^+^-Sal^-^ micelles at different pH.**

The synthesis of the biporous nanostructure involved using cetyltrimethylammonium chloride (CTAC) as a structure-directing surfactant, sodium salicylate (NaSal) as an auxiliary spacer, and triethanolamine (TEA) as a pH adjuster and catalyst to form pH-sensitive micelles^14,15,16,17^. At high pH, where OH^-^ predominates and Sal^-^ interacts less with CTA^+^, micelles exhibited a highly positive charge density, inducing intermicellar electrostatic repulsion and small pore formation in the PSNF. At low pH, the enhanced interaction between Sal- and CTA+ reduces charge density, leading to micelle aggregation, which serves as templates for large pores in BSNF.


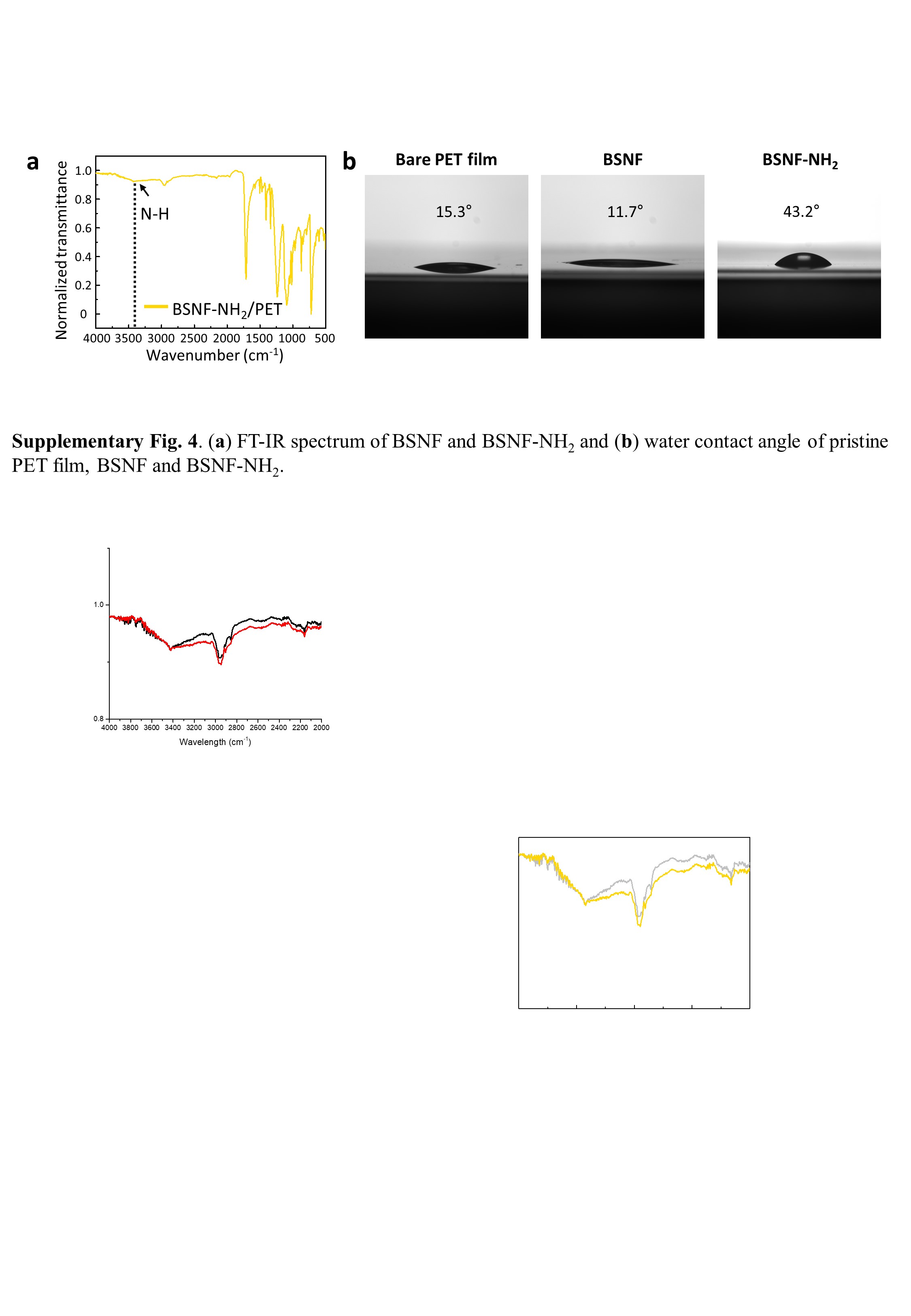


**Supplementary Fig. 2. Characterization of amine-modified surface.** **a**, FT-IR spectrum of BSNF-NH_2_. **b**, water contact angle of bare PET film, BSNF and BSNF-NH_2_. Fourier transform infrared (FT-IR) spectrum analysis revealed the presence of the 3350 cm^-1^ peak for the N-H group, along with an increase in the water contact angle of the BSNF following APDMS treatment, indicating amine functionalization of the BSNF. Source data are provided as a Source Data file.


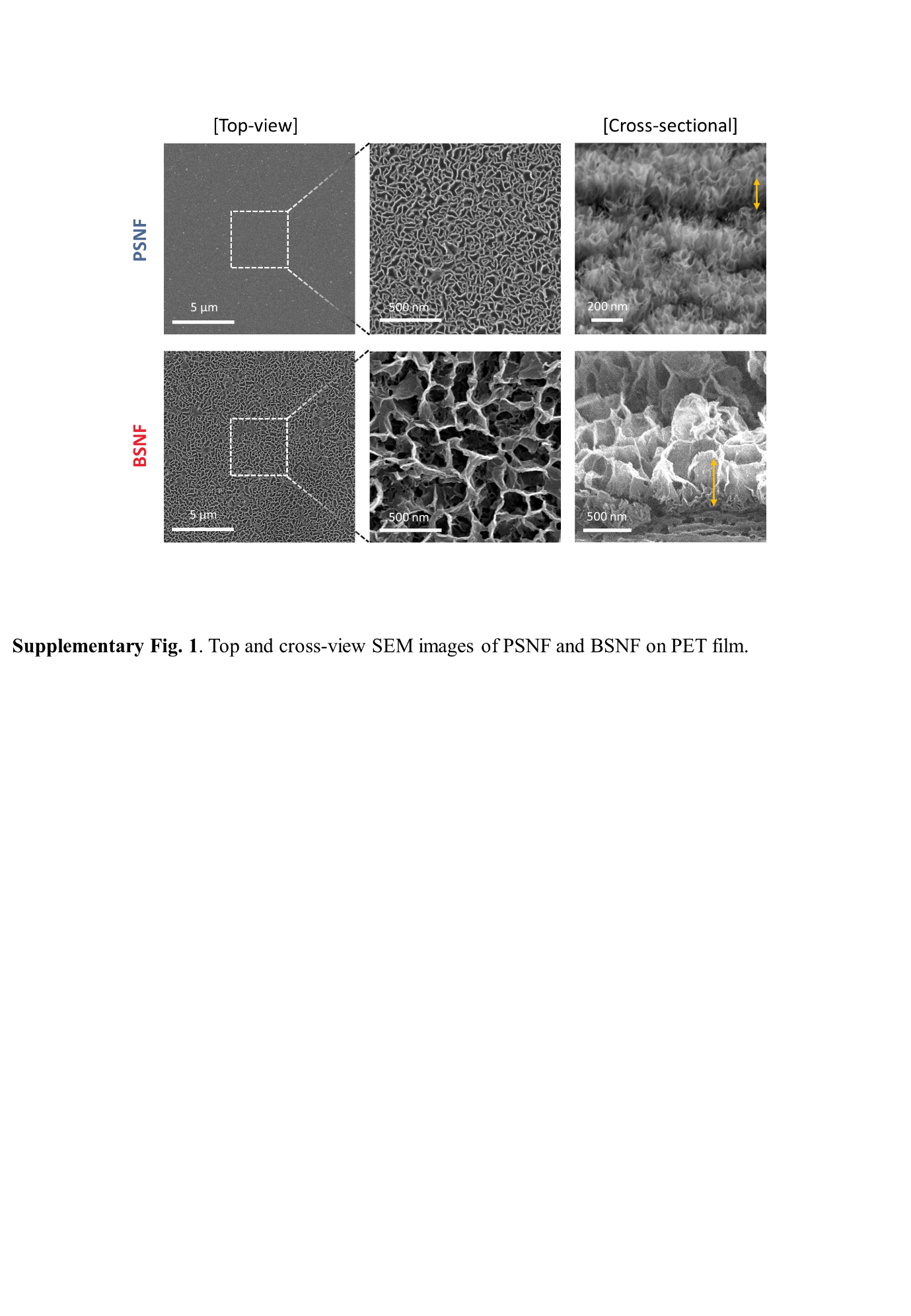


**Supplementary Fig. 3. SEM characterization of the nanofilm on the PET film.** Top-view and cross-sectional SEM images of PSNF and BSNF on the PET film. Data are representative of *n* = 3 independent experiments.


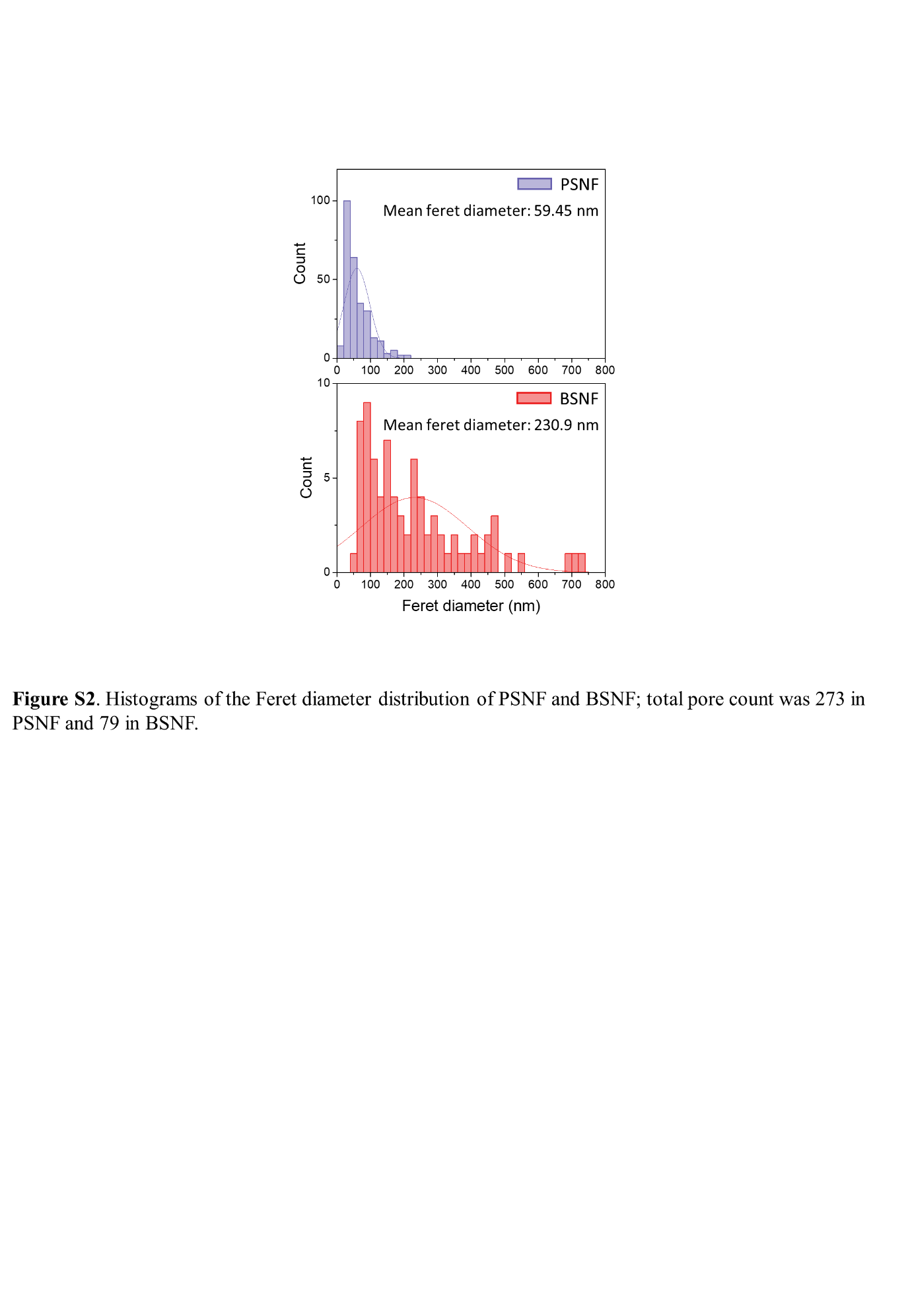


**Supplementary Fig. 4. Histograms of the Feret diameter distribution of PSNF and BSNF.** Total pore count was 273 in PSNF and 79 in BSNF. Source data are provided as a Source Data file.


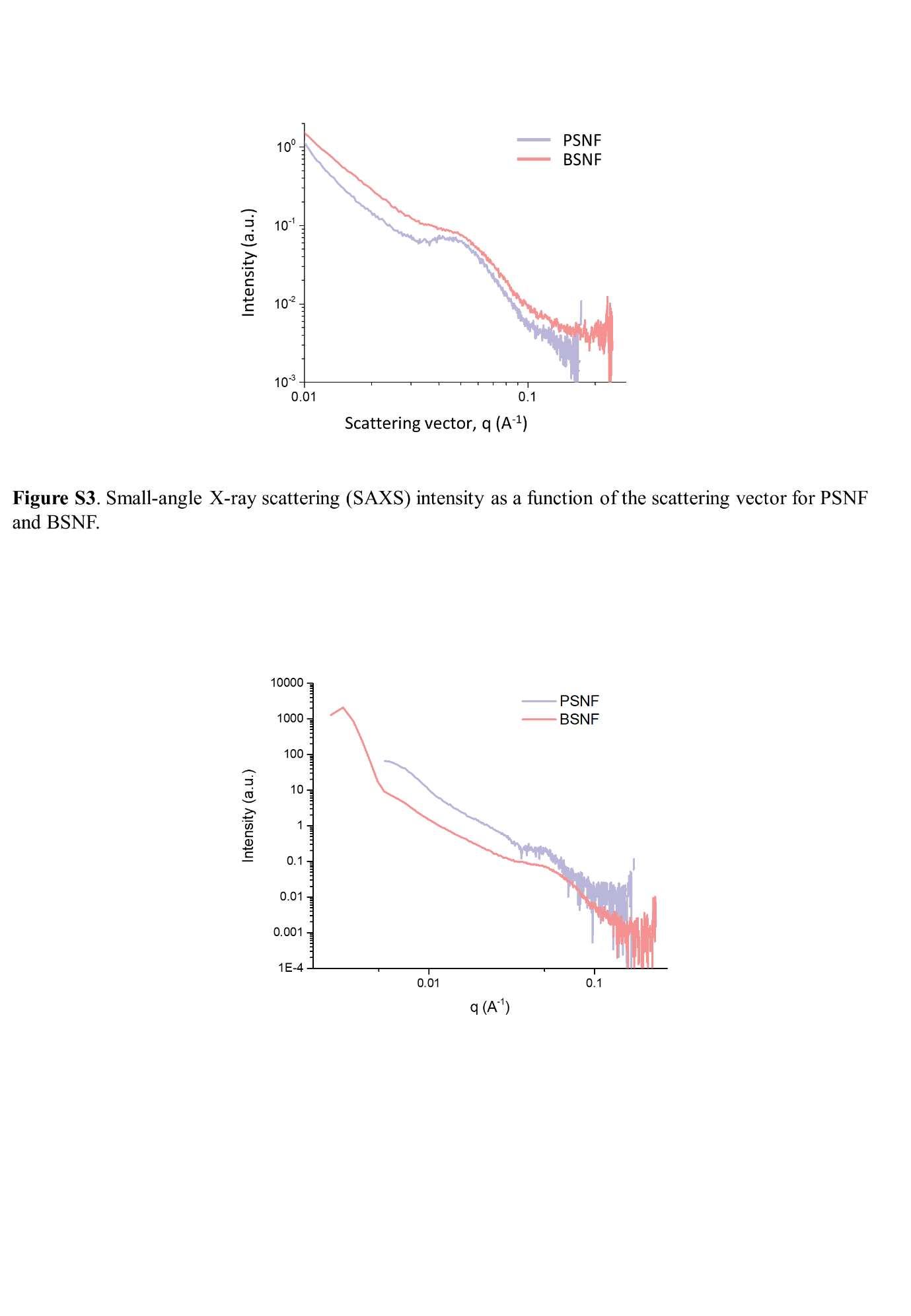


**Supplementary Fig. 5**. **Small-angle x-ray scattering (SAXS) analysis.** The graph shows intensity plotted against the scattering vector for PSNF and BSNF. a.u., arbitrary units. Source data are provided as a Source Data file.


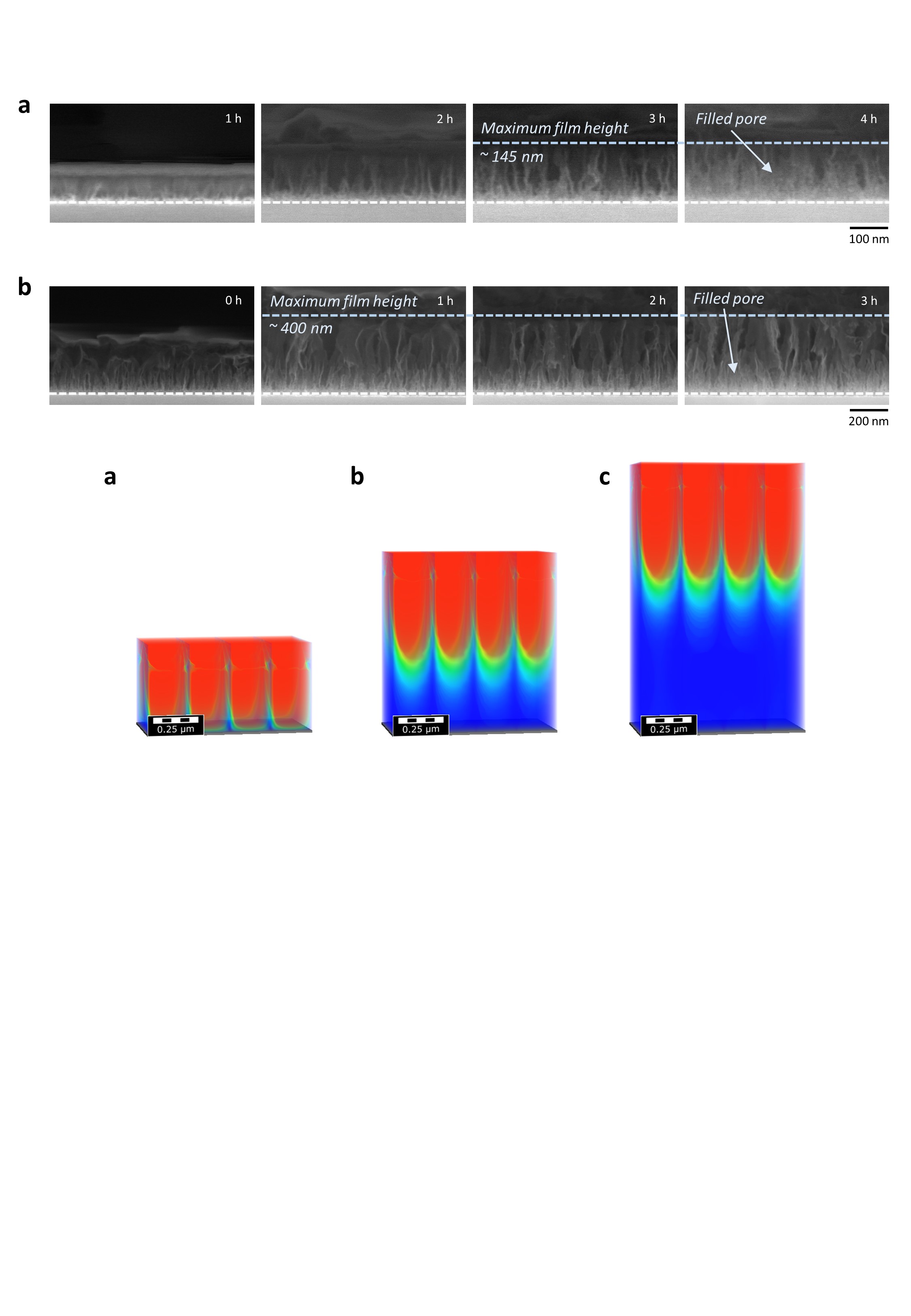


**Supplementary Fig. 6.** **Cross-sectional SEM images of a, PSNF and b, BSNF over reaction time.**

These nanostructures displayed gradual growth over the reaction time. However, the small pores in the first layer ceased growth after 3 hours, reaching a maximum height of about 145 nm. Subsequently, they began filling from the bottom, resulting in a reduction of the effective pore channel height. Similarly, the large pores in the second layer maintained their maximum height of 400 nm after 2 hours without further growth.


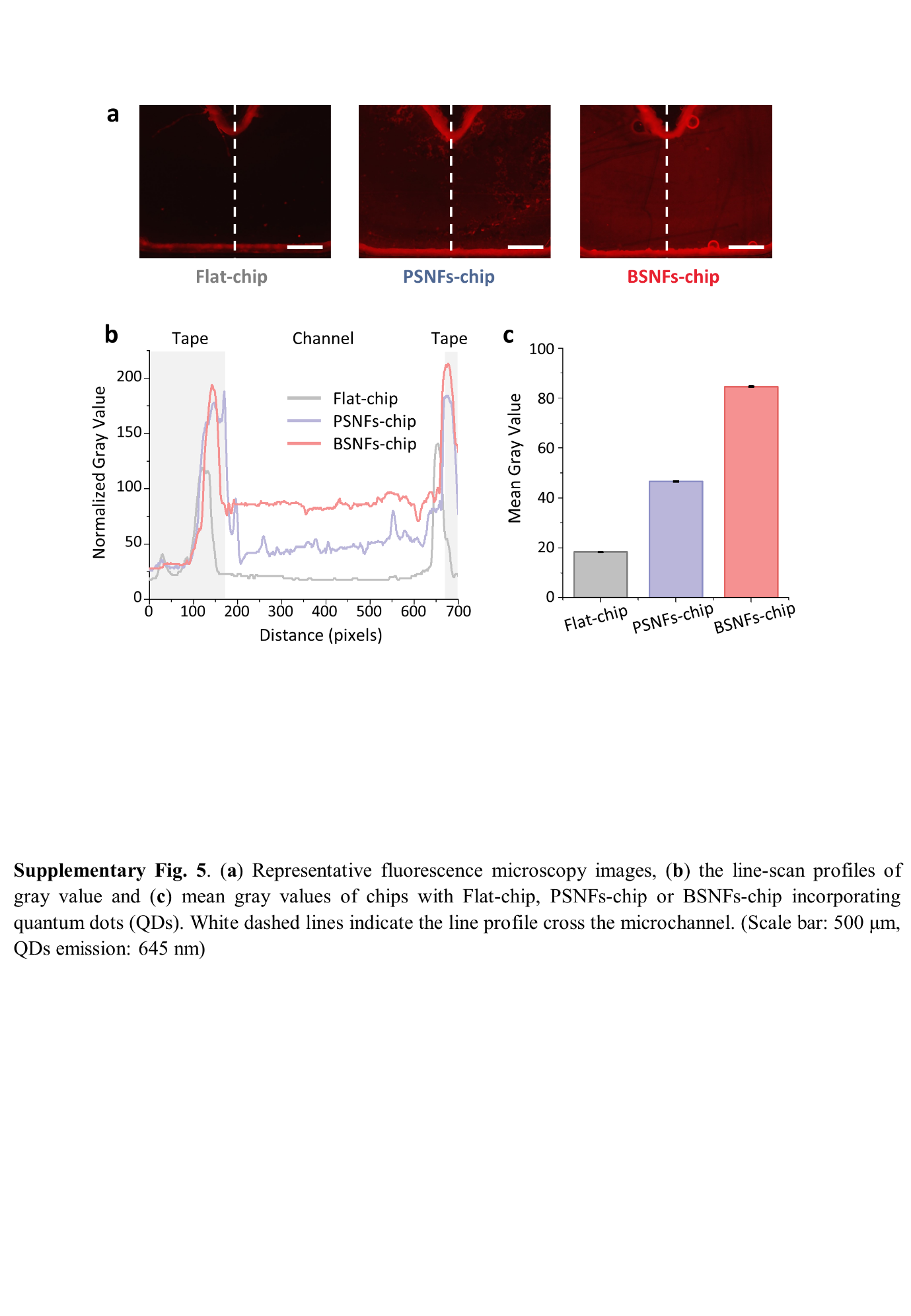


**Supplementary Fig. 7. Evaluation of the capture efficiency.** **a**, Representative fluorescence microscopy images, **b**, the line-scan profiles of gray value and **c**, mean gray values of chips with Flat-chip, PSNFs-chip or BSNFs-chip incorporating quantum dots (QDs). White dashed lines indicate the line profile cross the microchannel. (Scale bar: 500 μm, QDs emission: 645 nm). We assessed the mean fluorescence intensity resulting from QDs capture. Both the PSNFs- and BSNFs-chips exhibited higher fluorescence intensity due to their increased surface area compared to the Flat-chip, a conventional sample preparation chip with a flat surface. Source data are provided as a Source Data file.


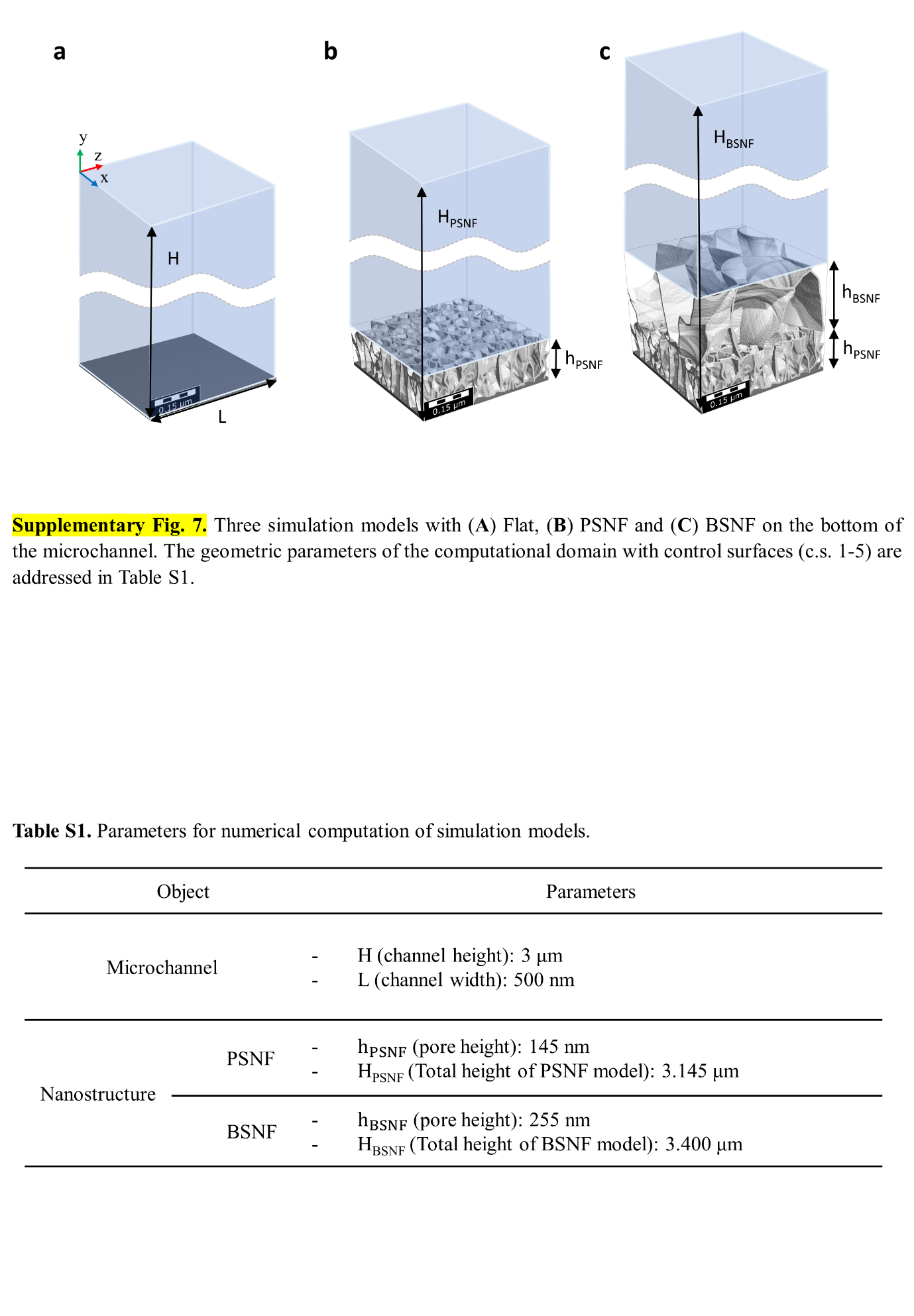


**Supplementary Fig. 8. Schematic illustrations for simulation model. a-c**, Three simulation models with (**a**), Flat, (**b**) PSNF and (**c**) BSNF on the bottom of the microchannel. The geometric parameters of the computational domain are addressed in Supplementary Table 1. In order to observe the flow in the nanostructure region, the height of the fluid channel was simulated to be 3 μm, which is a reduced scale (1/100) of the actual BSNFs-chip channel height of 300 μm, and then the images were cropped to a height of 600 nm.


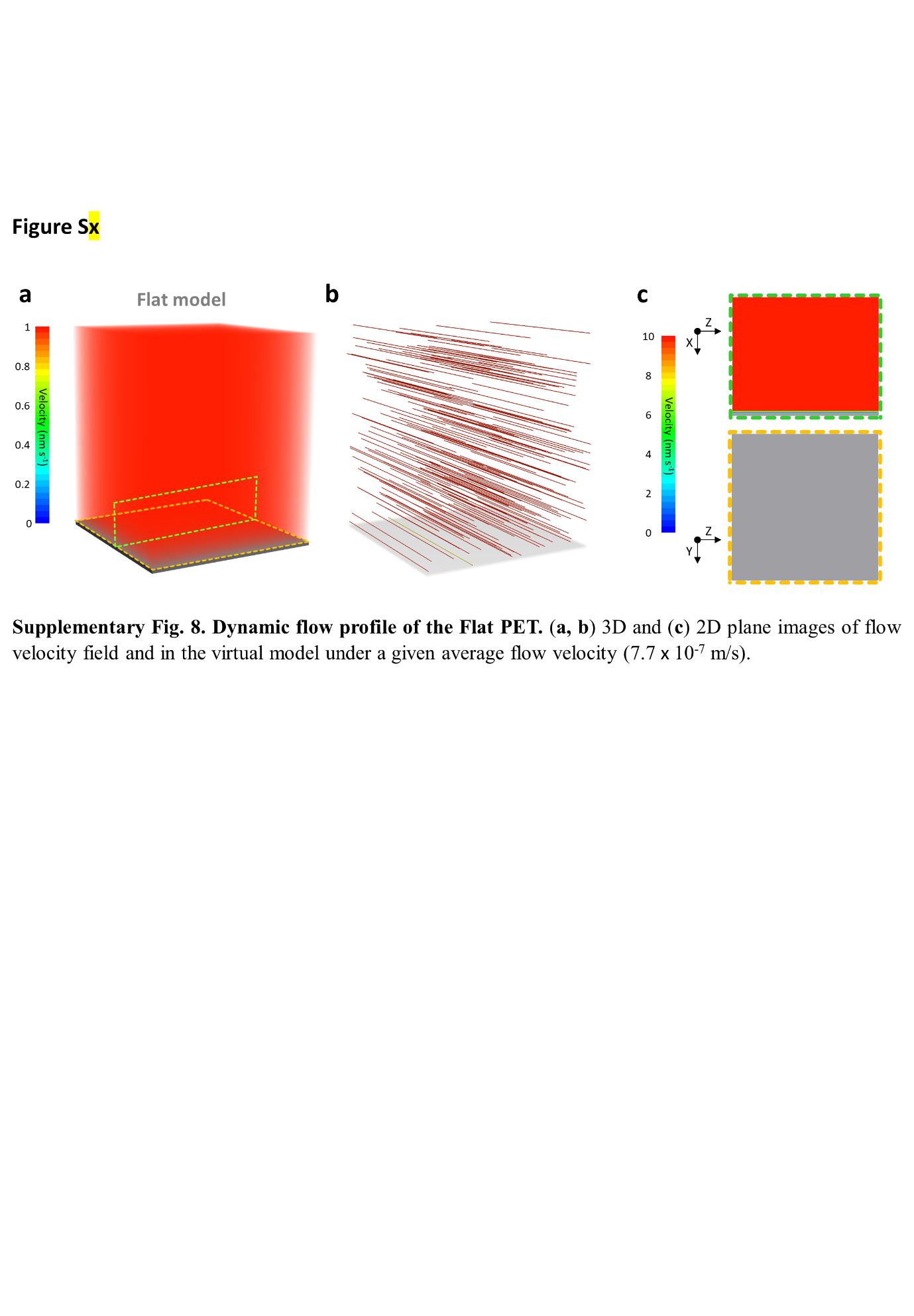


**Supplementary Fig. 9**. **Dynamic flow profile of the Flat PET.** **a**,**b**, 3D and **c**, 2D plane images of flow velocity field and in the virtual model under a given average flow velocity (7.7 x 10^-7^ m s^-1^).


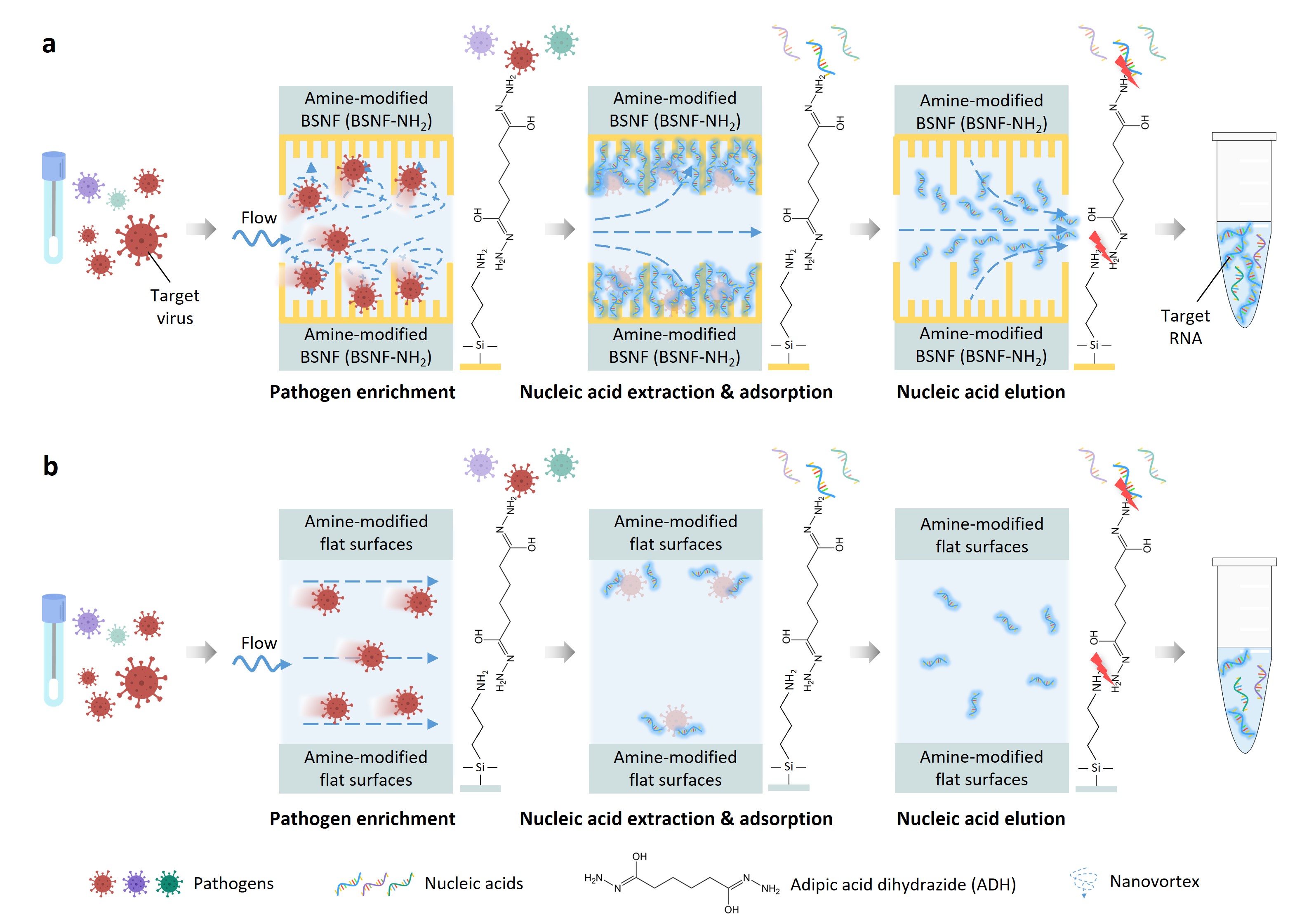


**Supplementary Fig.10**. **Schematic overviews of the sample preparation process of chips.** **a**, BSNFs-chip. **b**, Flat-chip. The amine-modified BSNFs and flat surfaces are designed to electrostatically capture a range of entities with a negative surface charge, including cells, bacteria, viruses, and vesicles.


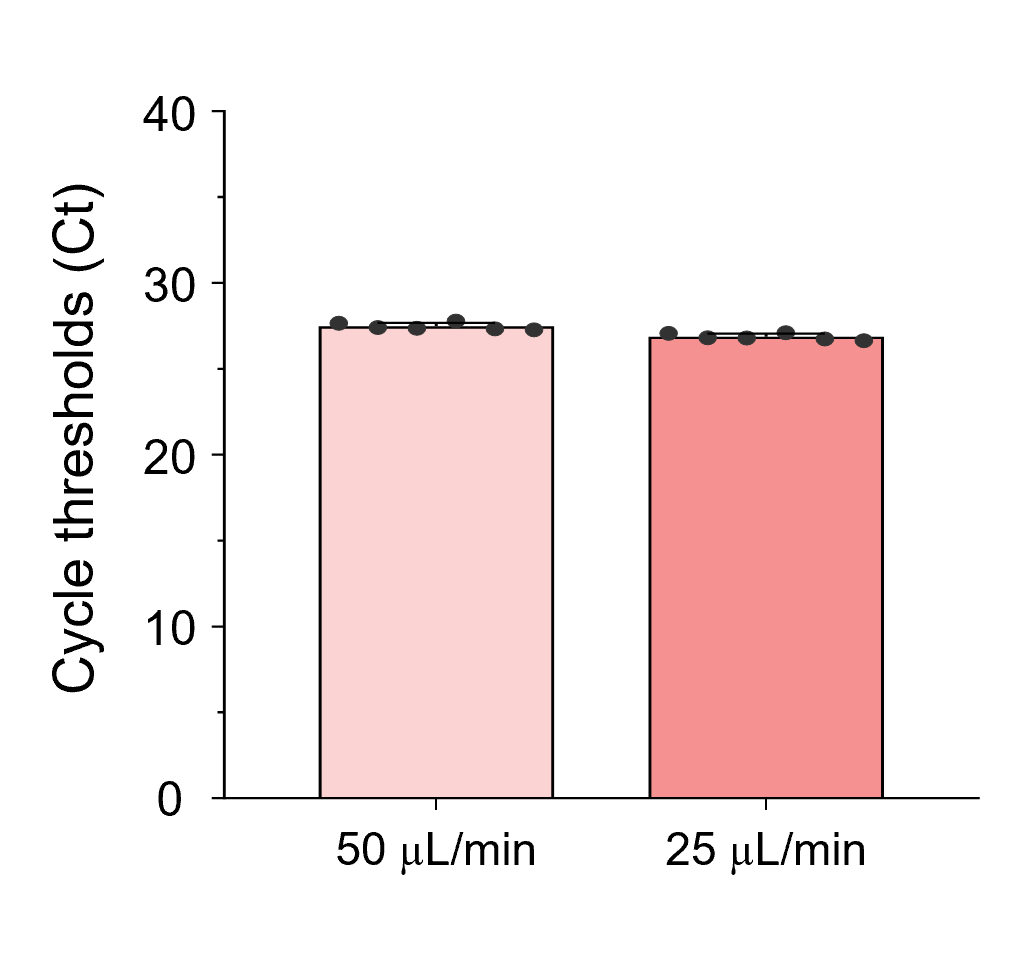


**Supplementary Fig. 11**. **NA elution flow rate test in the BSNFs-chip.** The flow rates tested were 50 µl min^-1^ and 25 µl min^-1^, with corresponding Ct values of 27.47 ± 0.19 and 26.86 ± 0.17, respectively. *n* = 6 biologically independent experiments per group. Data are presented as mean values ± SD. Source data are provided as a Source Data file.


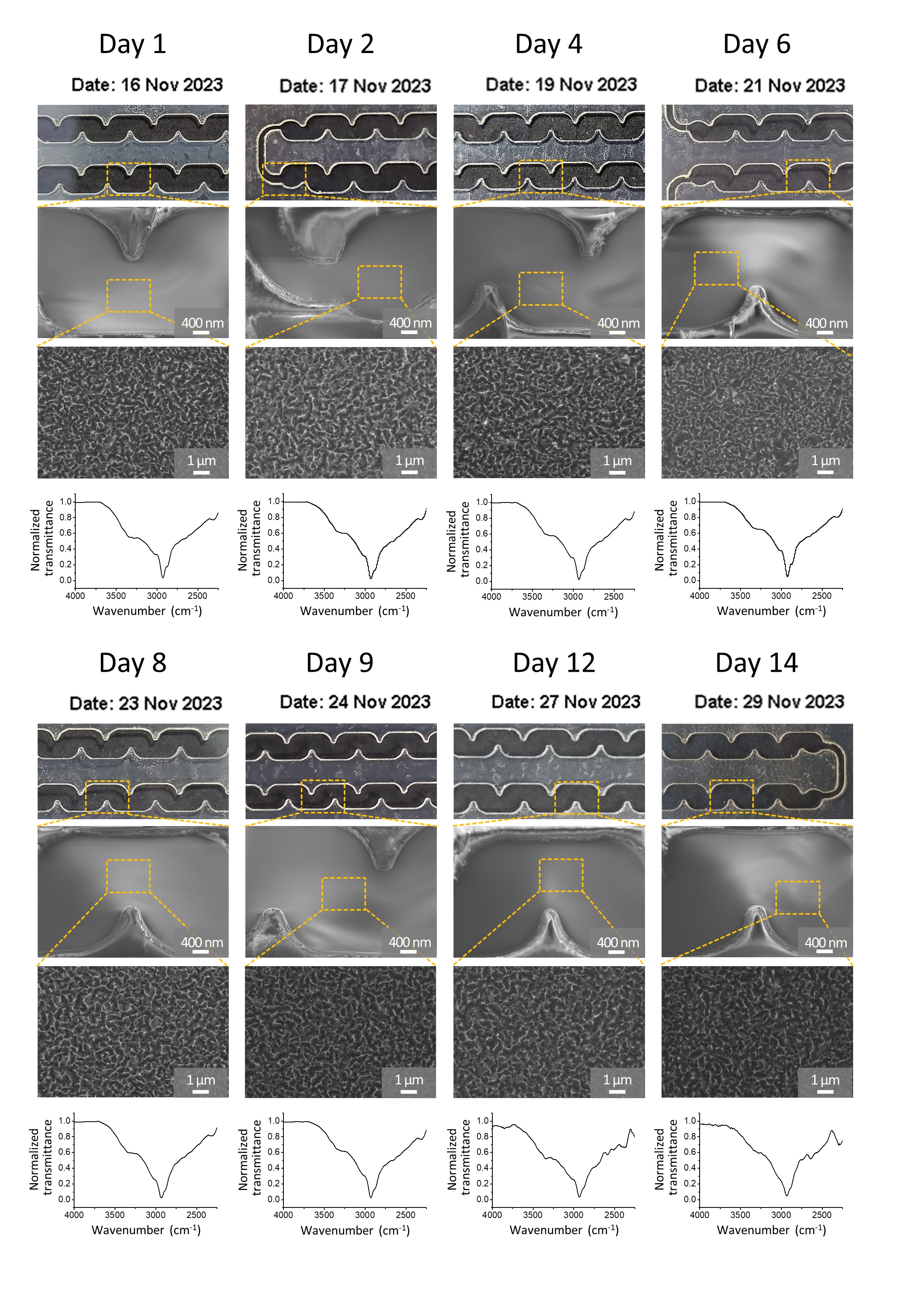


**Supplementary Fig. 12. Stability of the BSNFs-chip.** SEM images and FT-IR spectrums showing the stability of the BSNFs-chip over a two-week period immediately following chip fabrication. Source data are provided as a Source Data file.

The BSNFs-chip was utilized underwent prolonged storage at room temperature post-fabrication. These conditions were consistent across experiments, notably in sample preparations for PCR test and LRET assay.


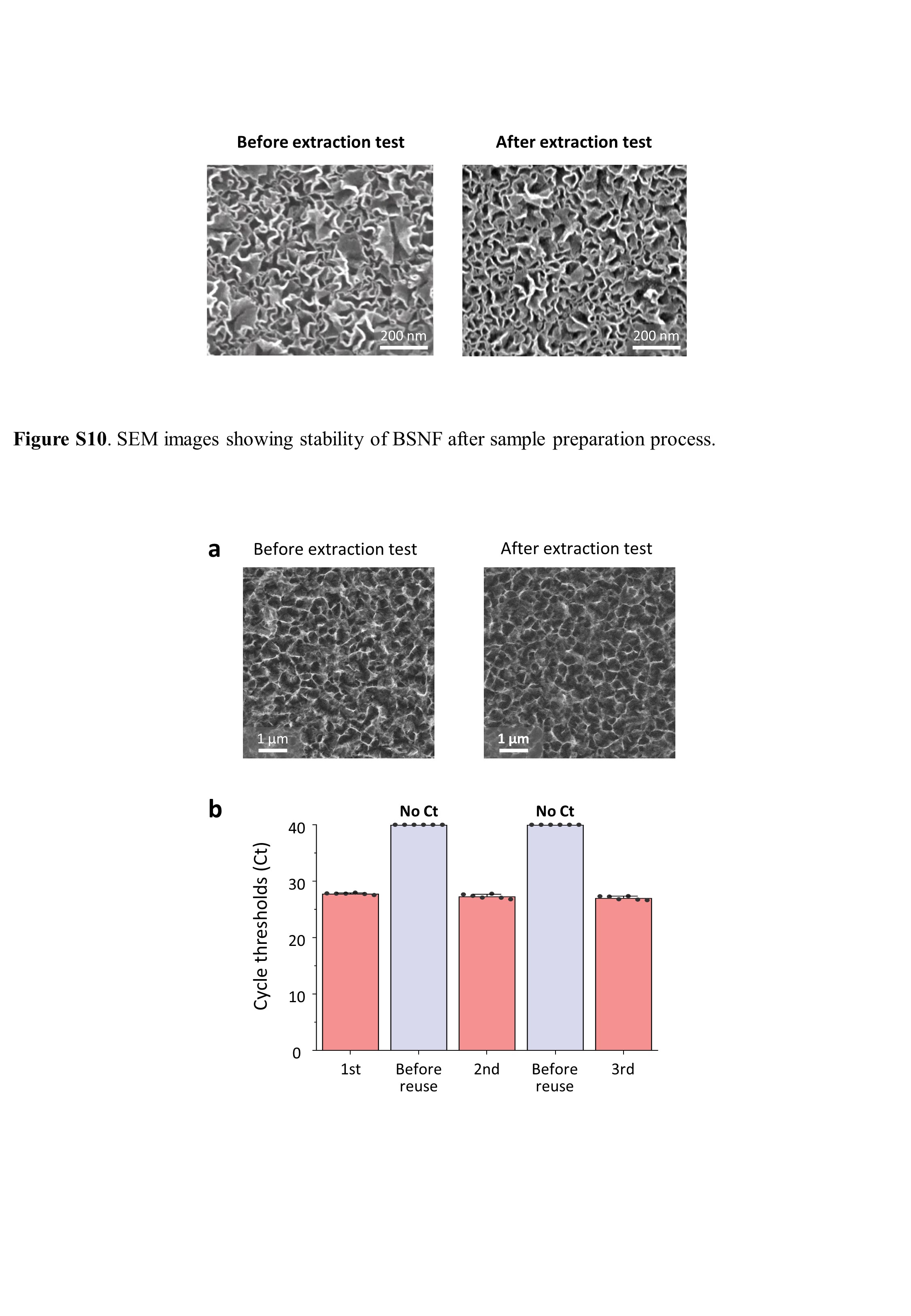


**Supplementary Fig. 13. Verification of structural stability and reusability.** **a**, SEM images showing stability of BSNF after sample preparation process. **b**, Analysis of structural robustness and reusability in pathogen and NA enrichment/isolation using the BSNFs-chip. *n* = 6 biologically independent experiments per group. Data are presented as mean values ± SD. Source data are provided as a Source Data file.


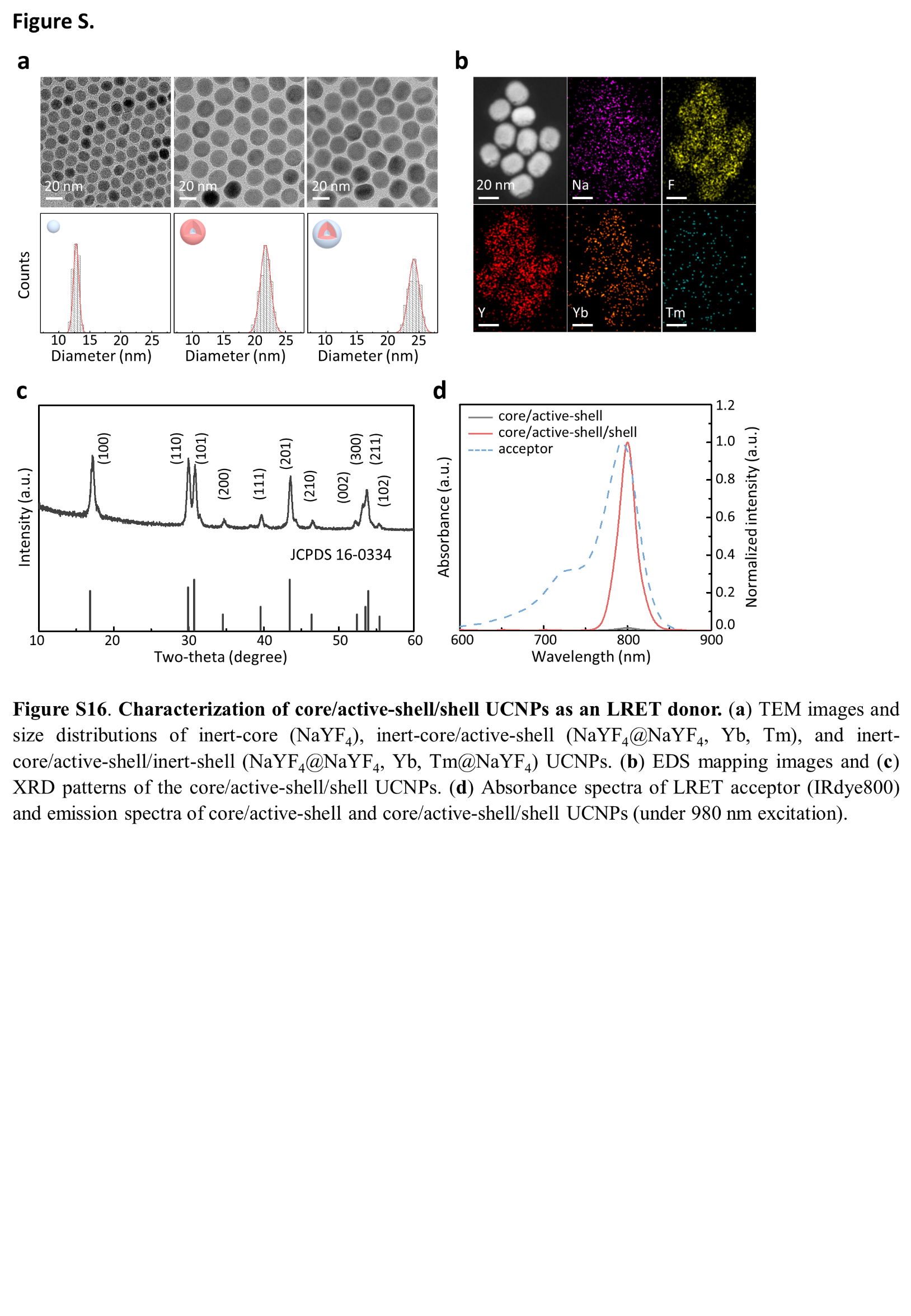


**Supplementary Fig. 14**. **Characterization of core/active-shell/shell LnNPs as an LRET donor. a**, TEM images and size distributions of inert-core (NaYF_4_), inert-core/active-shell (NaYF_4_@NaYF_4_, Yb, Tm), and inert-core/active-shell/inert-shell (NaYF_4_@NaYF_4_, Yb, Tm@NaYF_4_) LnNPs. **b**,**c** EDS mapping images (**b**) and XRD patterns (**c**) of the core/active-shell/shell LnNPs. **d**, Absorbance spectra of LRET acceptor (IRdye800) and emission spectra of core/active-shell and core/active-shell/shell LnNPs (under 980 nm excitation). a.u., arbitrary units. Source data are provided as a Source Data file.


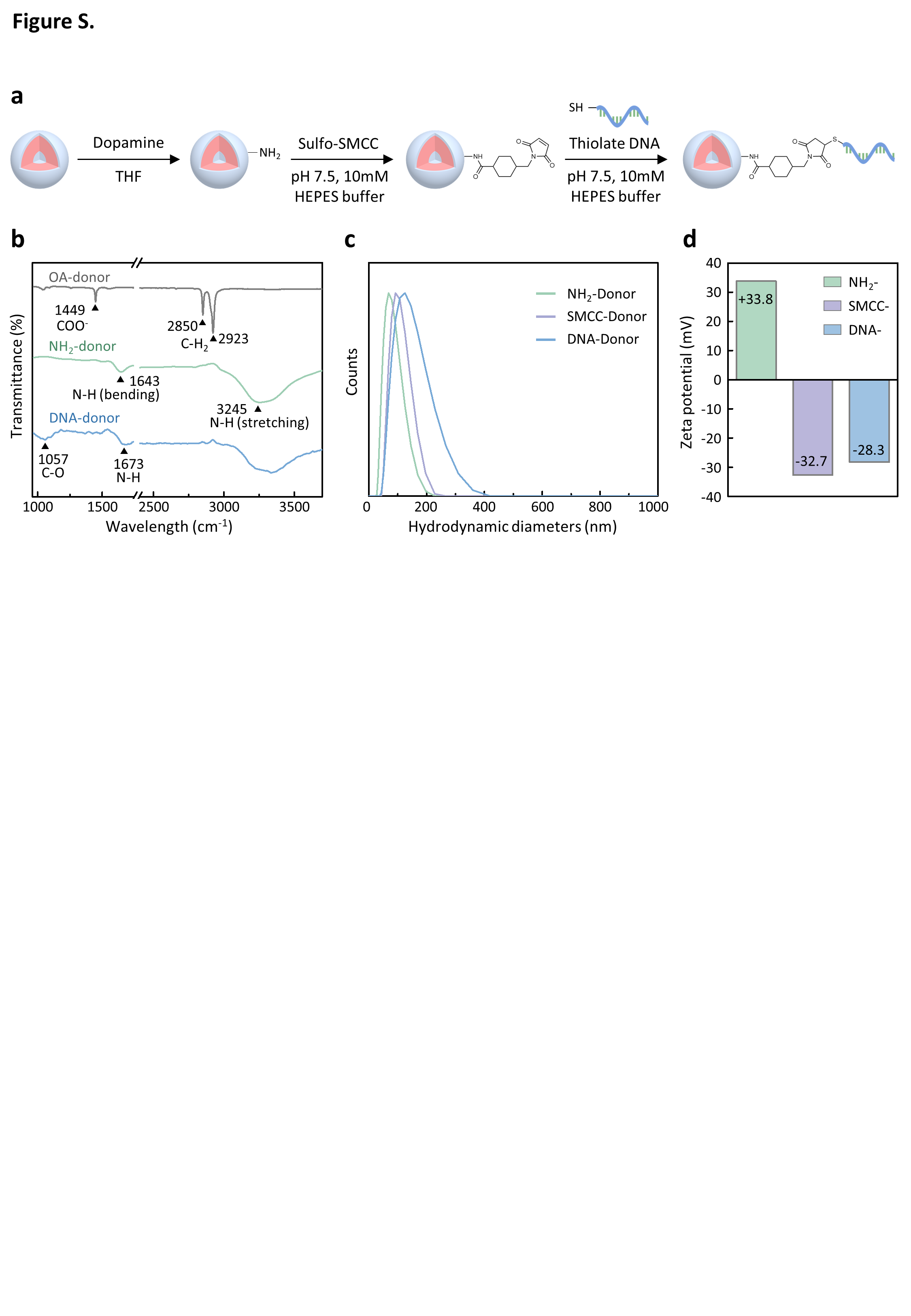


**Supplementary Fig. 15**. **Surface modification of the LRET donor. a**, Schematic illustration of conjugation path of capture DNA and LnNP. **b**, FT-IR spectra of oleic acid capped, NH_2_-, and DNA-donor. **c**,**d**, Dynamic light scattering spectra (**c**) and zeta potentials (**d**) of NH_2_-, SMCC-, and DNA-donor. Source data are provided as a Source Data file.


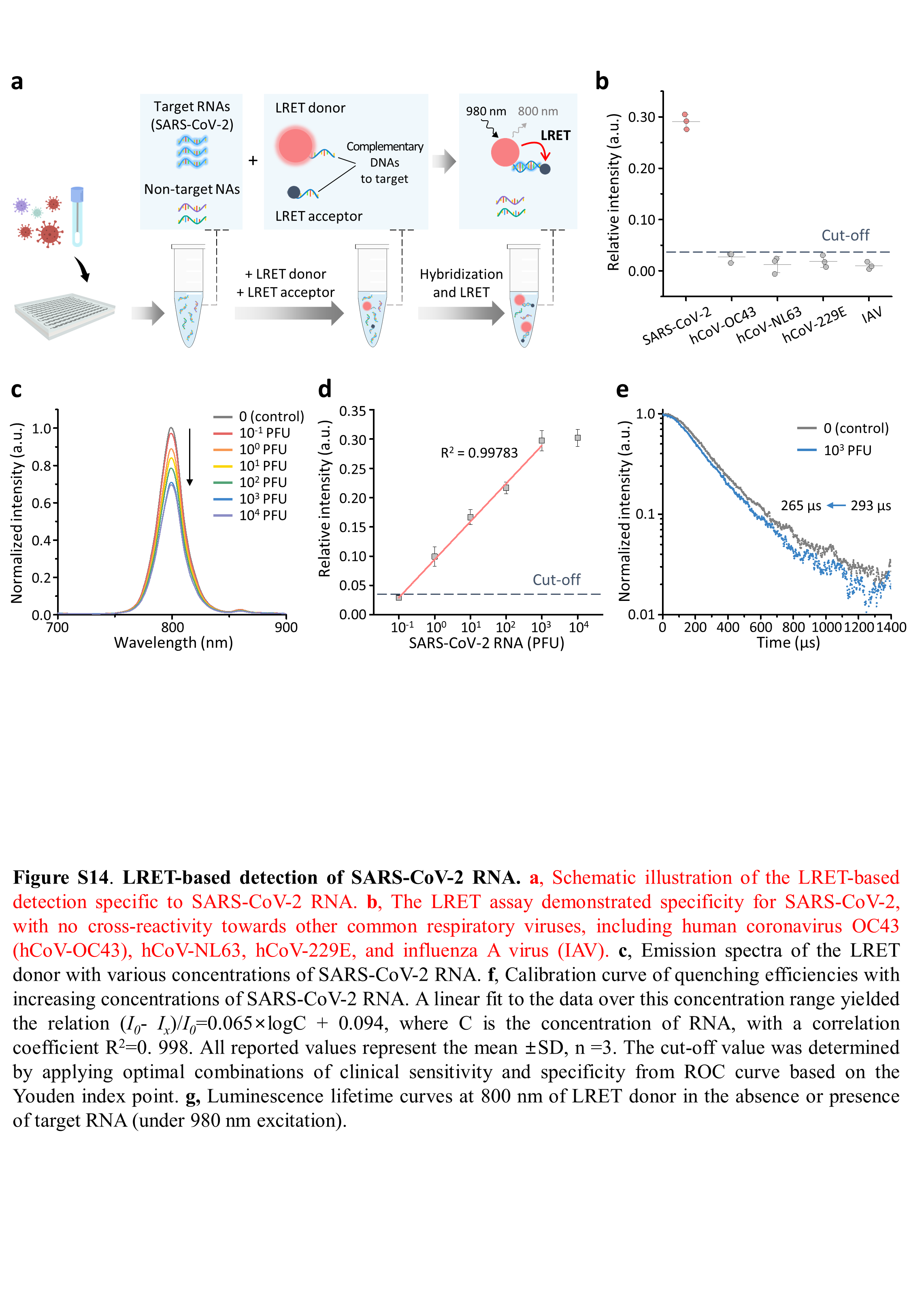


**Supplementary Fig. 16**. **LRET-based detection of SARS-CoV-2 RNA. a**, Schematic illustration of the LRET-based detection specific to SARS-CoV-2 RNA. **b**, The LRET assay demonstrated specificity for SARS-CoV-2, with no cross-reactivity towards other common respiratory viruses, including human coronavirus OC43 (hCoV-OC43), hCoV-NL63, hCoV-229E, and influenza A virus (IAV). Data are expressed as mean ± SD (*n* = 3 independent experiments). a.u., arbitrary units. **c**, Emission spectra of the LRET donor with various concentrations of SARS-CoV-2 RNA. **d**, Calibration curve of quenching efficiencies with increasing concentrations of SARS-CoV-2 RNA. A linear fit to the data over this concentration range yielded the relation (*I_0_*- *I_x_*)/*I_0_*=0.065×logC + 0.094, where C is the concentration of RNA, with a correlation coefficient R^2^=0. 998. All reported values represent the mean ±SD, *n* =3 independent experiments. The cut-off value was determined by applying optimal combinations of clinical sensitivity and specificity from ROC curve based on the Youden index point. **e,** Luminescence lifetime curves at 800 nm of LRET donor in the absence or presence of target RNA (under 980 nm excitation). Source data are provided as a Source Data file.


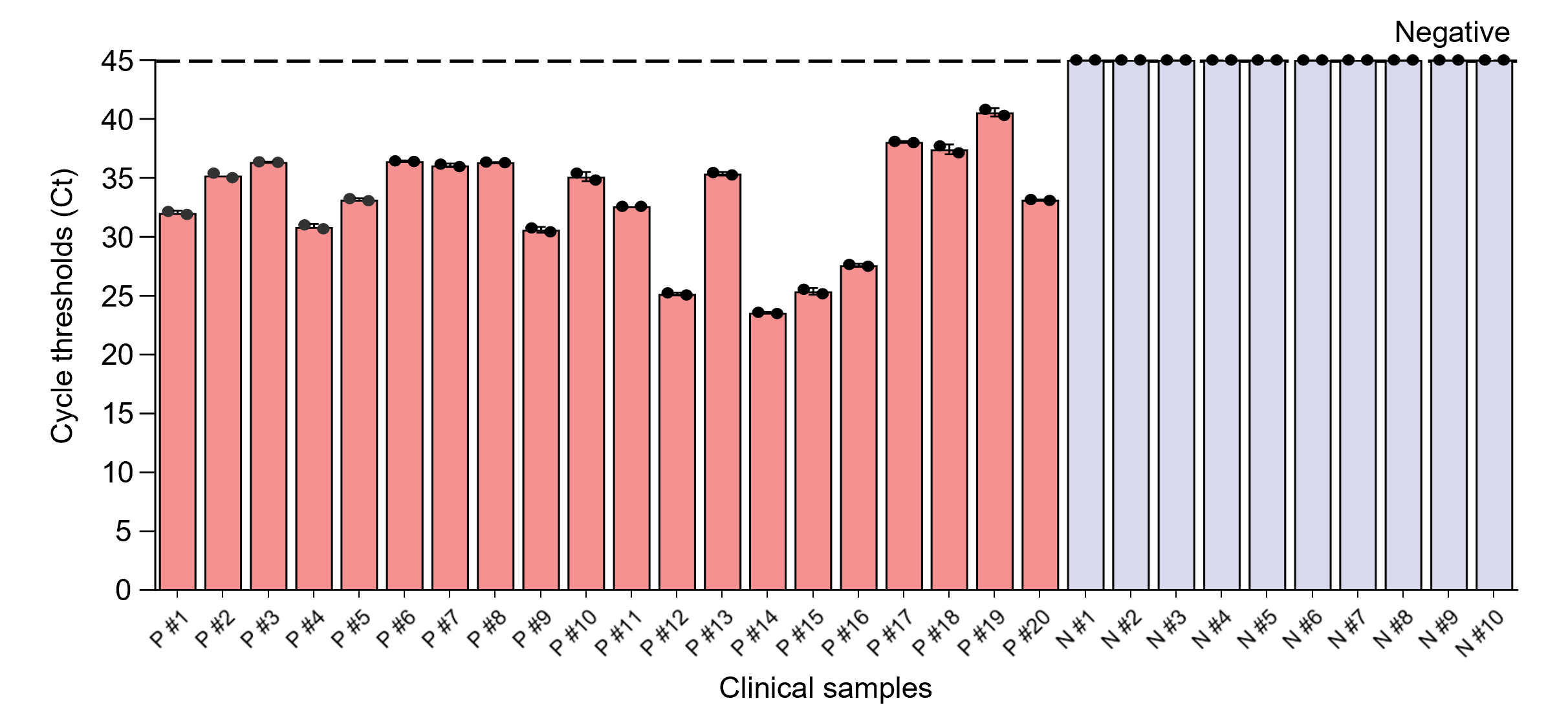


**Supplementary Fig. 17**. **qRT-PCR detection of SARS-CoV-2 RNA in clinical samples.** qRT-PCR detection of SARS-CoV-2 RNA extracted from the BSNFs-chip using 30 nasopharyngeal (NP) swab samples identified as confirmed cases by clinical definitions. *n* = 2 biologically independent experiments per group. Data are presented as mean values ± SD. Source data are provided as a Source Data file.

**
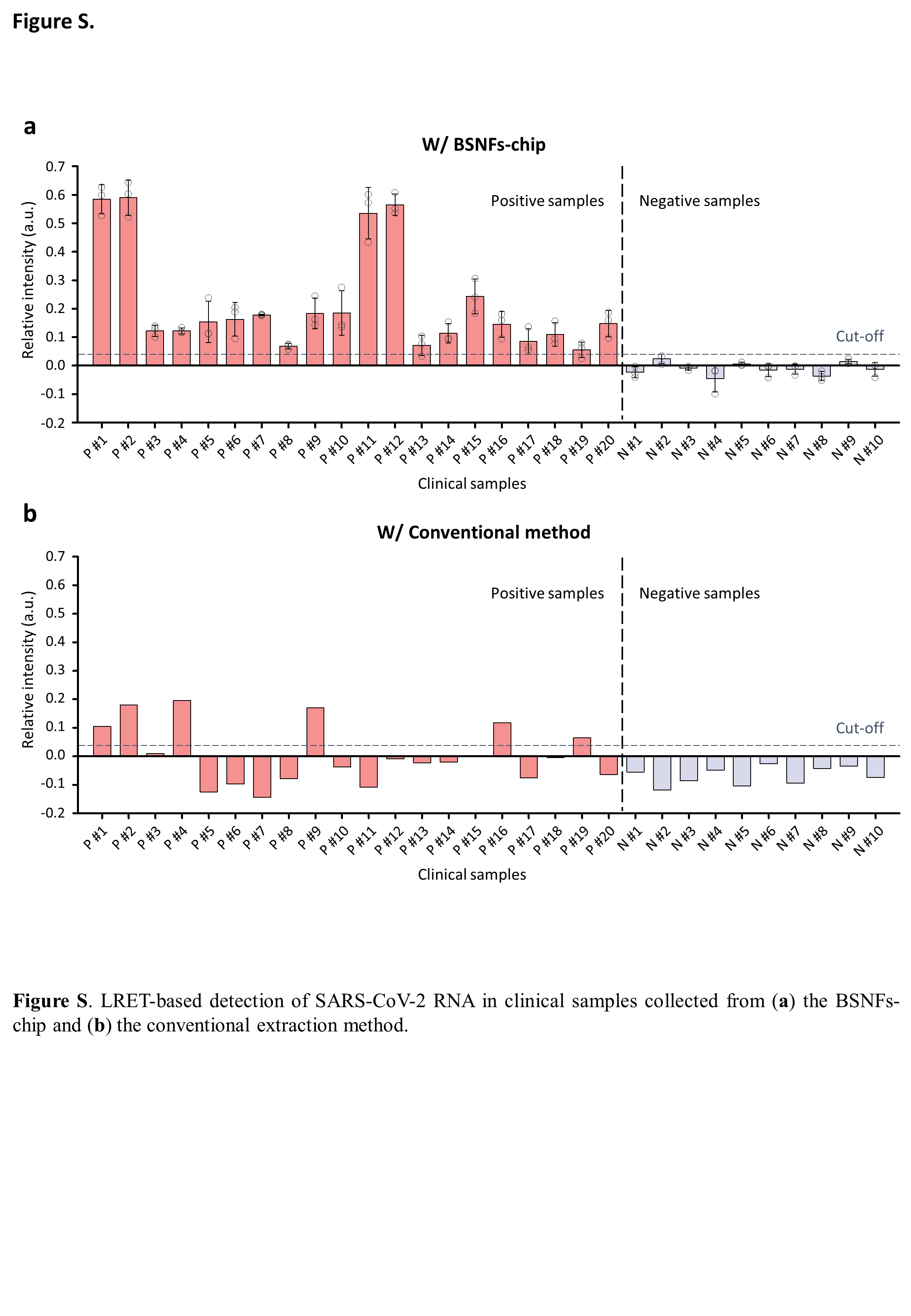
**

**Supplementary Fig. 18. LRET assay for SARS-CoV-2 RNA in clinical samples.** **a**,**b**, LRET assay for SARS-CoV-2 RNA in clinical samples collected from (**a**) the BSNFs- chip and (**b**) the conventional extraction method. The reported values in (a) represent the mean ±SD, *n* =3 independent experiments. a.u., arbitrary units. Source data are provided as a Source Data file.

**Supplementary Table 1. Parameters for numerical computation of simulation models.**

| Object | | Parameters |
| --- | --- | --- |
| Microchannel | | - H (channel height): 3 μm - L (channel width): 500 nm |
| Nanostructure | PSNF | - h_PSNF_ (pore height): 145 nm - H_PSNF_ (Total height of PSNF model): 3.145 μm |
|  | BSNF | - h_BSNF_ (pore height): 255 nm - H_BSNF_ (Total height of BSNF model): 3.400 μm |

**Supplementary Table 2. Primer and probe sets used in this study.**

| qPCR-based  methods | Target  (Gene) | Sequence (5'-3') | | Mer | Modification |
| --- | --- | --- | --- | --- | --- |
| qPCR | HCT116  (ACTB) | Forward | CTG GAA CGG TGA AGG TGA CA | 20 |  |
|  |  | Reverse | AAG GGA CTT CCT GTA ACA ATG CA | 23 |  |
| qRT-PCR | HCT116  (18s rRNA) | Forward | GCT TAA TTT GAC TCA ACA CGG GA | 23 |  |
|  |  | Reverse | AGC TAT CAA TCT GTC AAT CCT GTC | 24 |  |
|  | SARS-CoV-2  (S gene) | Forward | TCC GCA TCA TTT TCC ACT TTT AA | 23 |  |
|  |  | Reverse | TAA CGC AGC CTG TAA AAT CAT CTG | 24 |  |
|  |  | Probe | AAG TCA GAC AAA TCG CTC CAG GGC AAA | 27 | Cy5 (5')  BHQ-2 (3') |

**Supplementary Table 3. Raw qPCR data showing the LODs for genomic DNA using the HCT116 cell.**

| Raw data of qPCR for genomic DNA  (ACTB gene from HCT116 cell) | | | | | | | |
| --- | --- | --- | --- | --- | --- | --- | --- |
| Type | Concentration  (1 x 10^N^ Cell/mL) | #1 | #2 | #3 | #4 | Mean | STDEV |
| Conventional  method | 4 | 31.18 | 31.03 | 31.07 | . | 31.09 | 0.06 |
|  | 3 | 34.28 | 34.17 | 34.11 | . | 34.19 | 0.07 |
|  | 2 | 40 | 40 | 40 | . | . | . |
|  | 1 | 40 | 40 | 40 | . | . | . |
|  | 0 | 40 | 40 | 40 | . | . | . |
| Flat-chip | 4 | 28.43 | 28.09 | 27.88 | 28.32 | 28.18 | 0.21 |
|  | 3 | 32.47 | 31.73 | 31.62 | 31.83 | 31.91 | 0.33 |
|  | 2 | 36.03 | 35.50 | 35.42 | 35.79 | 35.69 | 0.24 |
|  | 1 | 40 | 40 | 40 | 40 | . | . |
|  | 0 | 40 | 40 | 40 | 40 | . | . |
| PSNFs-chip | 4 | 27.88 | 28.06 | 28.30 | 28.12 | 28.09 | 0.15 |
|  | 3 | 31.82 | 31.86 | 31.56 | 31.61 | 31.71 | 0.13 |
|  | 2 | 34.63 | 34.49 | 35.03 | 35.14 | 34.82 | 0.27 |
|  | 1 | 38.42 | 38.44 | 38.17 | 38.23 | 38.32 | 0.12 |
|  | 0 | 40 | 40 | 40 | 40 | . | . |
| BSNFs-chip | 4 | 27.85 | 27.81 | 27.89 | 27.56 | 27.78 | 0.13 |
|  | 3 | 31.61 | 31.39 | 31.58 | 31.55 | 31.53 | 0.08 |
|  | 2 | 34.77 | 34.74 | 34.53 | 34.34 | 34.60 | 0.17 |
|  | 1 | 37.85 | 37.93 | 37.61 | 37.68 | 37.77 | 0.13 |
|  | 0 | 40 | 40 | 40 | 40 | . | . |

**Supplementary Table 4. Raw qRT-PCR data showing the LODs for genomic RNA using the HCT116 cell.**

| Raw data of qRT-PCR for genomic RNA  (18S rRNA gene from HCT116 cell) | | | | | | | |
| --- | --- | --- | --- | --- | --- | --- | --- |
| Type | Concentration  (1 x 10^N^ Cell/mL) | #1 | #2 | #3 | #4 | Mean | STDEV |
| Conventional  method | 4 | 31.76 | 31.60 | 31.49 | . | 31.62 | 0.11 |
|  | 3 | 34.95 | 35.17 | 34.76 | . | 34.96 | 0.17 |
|  | 2 | 40 | 40 | 40 | . | . | . |
|  | 1 | 40 | 40 | 40 | . | . | . |
|  | 0 | 40 | 40 | 40 | . | . | . |
| Flat-chip | 4 | 28.81 | 28.79 | 28.91 | 29.09 | 28.90 | 0.12 |
|  | 3 | 32.23 | 32.22 | 32.36 | 32.47 | 32.32 | 0.10 |
|  | 2 | 35.89 | 35.92 | 36.14 | 36.16 | 36.03 | 0.12 |
|  | 1 | 40 | 40 | 40 | 40 | . | . |
|  | 0 | 40 | 40 | 40 | 40 | . | . |
| PSNFs-chip | 4 | 29.01 | 28.87 | 28.43 | 28.47 | 28.70 | 0.25 |
|  | 3 | 31.94 | 31.99 | 32.23 | 32.38 | 32.14 | 0.18 |
|  | 2 | 35.79 | 35.76 | 35.96 | 35.84 | 35.84 | 0.08 |
|  | 1 | 40 | 40 | 40 | 40 | . | . |
|  | 0 | 40 | 40 | 40 | 40 | . | . |
| BSNFs-chip | 4 | 28.64 | 28.57 | 28.42 | 28.29 | 28.48 | 0.14 |
|  | 3 | 31.78 | 31.99 | 32.26 | 32.01 | 32.01 | 0.17 |
|  | 2 | 35.41 | 35.32 | 35.59 | 35.43 | 35.44 | 0.10 |
|  | 1 | 38.72 | 38.84 | 38.61 | 38.56 | 38.68 | 0.11 |
|  | 0 | 40 | 40 | 40 | 40 | . | . |

**Supplementary Table 5. Raw qRT-PCR data showing the LODs for viral RNA using the SARS-CoV-2 culture fluid.**

| Raw data of qRT-PCR for viral RNA  (S gene from SARS-CoV-2) | | | | | | |
| --- | --- | --- | --- | --- | --- | --- |
| Type | Concentration  (0.96 x 10^N^ PFU/mL) | #1 | #2 | #3 | Mean | STDEV |
| Conventional  method | 4 | 30.07 | 30.07 | 30.41 | 30.18 | 0.16 |
|  | 3 | 33.37 | 33.67 | 33.82 | 33.62 | 0.19 |
|  | 2 | 36.83 | 36.99 | 37.45 | 37.09 | 0.26 |
|  | 1 | 45 | 45 | 45 | . | . |
|  | 0 | 45 | 45 | 45 | . | . |
|  | −1 | 45 | 45 | 45 | . | . |
| Flat-chip | 4 | 27.55 | 27.69 | 27.72 | 27.65 | 0.07 |
|  | 3 | 30.84 | 30.60 | 30.75 | 30.73 | 0.10 |
|  | 2 | 34.47 | 34.41 | 34.29 | 34.39 | 0.07 |
|  | 1 | 36.91 | 36.88 | 36.99 | 36.93 | 0.05 |
|  | 0 | 45 | 45 | 45 | . | . |
|  | −1 | 45 | 45 | 45 | . | . |
| PSNFs-chip | 4 | 27.24 | 27.31 | 27.69 | 27.41 | 0.20 |
|  | 3 | 30.51 | 30.59 | 30.39 | 30.50 | 0.08 |
|  | 2 | 33.72 | 34.04 | 34.08 | 33.95 | 0.16 |
|  | 1 | 36.69 | 36.64 | 37.01 | 36.78 | 0.16 |
|  | 0 | 45 | 45 | 45 | . | . |
|  | −1 | 45 | 45 | 45 | . | . |
| BSNFs-chip | 4 | 26.99 | 26.80 | 26.93 | 26.91 | 0.08 |
|  | 3 | 30.03 | 30.16 | 30.15 | 30.11 | 0.06 |
|  | 2 | 33.41 | 32.92 | 33.05 | 33.13 | 0.21 |
|  | 1 | 36.81 | 36.50 | 36.36 | 36.56 | 0.19 |
|  | 0 | 40.25 | 40.29 | 40.34 | 40.29 | 0.04 |
|  | −1 | 45 | 45 | 45 | . | . |

**Supplementary Table 6. The oligo sequences used in the LRET assay for SARS-CoV-2 S gene detection.**

|  | Sequence (5'-3') | Mer | Modification |
| --- | --- | --- | --- |
| DNA oligo for LRET donor | AAG TGG AAA ATG ATG CGG | 20 | Thiol (5’) |
| DNA oligo for LRET acceptor | AGA CAC TCC ATA ACA CTT AA | 18 | IR800 (5’) |
| Target site | CCG CAU CAU UUU CCA CUU UUA AGU GUU AUG GAG UGU CU | 38 |  |

**Supplementary References**

1. Shen, J. *et al.* Tunable Near Infrared to Ultraviolet Upconversion Luminescence Enhancement in (alpha-NaYF4:Yb,Tm)/CaF2 Core/Shell Nanoparticles for In situ Real-time Recorded Biocompatible Photoactivation. *Small* **9**, 3213-3217 (2013).

2. Du, Z., Gao, N., Wang, X. H., Ren, J. S. & Qu, X. G. Near-Infrared Switchable Fullerene-Based Synergy Therapy for Alzheimer's Disease. *Small* **14**, (2018).

3. Kang, D., Jeon, E., Kim, S. & Lee, J. S. Lanthanide-Doped Upconversion Nanomaterials: Recent Advances and Applications. *Biochip J.* **14**, 124-135 (2020).

4. Wen, S. H. *et al.* Advances in highly doped upconversion nanoparticles. *Nat. Commun.* **9**, (2018).

5. Kim, S. *et al.* On-Site Remote Monitoring System with NIR Signal-Based Detection of Infectious Disease Virus in Opaque Salivary Samples. *Acs Sensors* **8**, 1299-1307 (2023).

6. Kang, D. *et al.* A local water molecular-heating strategy for near-infrared long-lifetime imaging-guided photothermal therapy of glioblastoma. *Nat. Commun.* **14**, (2023).

7. Kang, D. *et al.* An NIR dual-emitting/absorbing inorganic compact pair: A self-calibrating LRET system for homogeneous virus detection. *Biosens. Bioelectron.* **190**, (2021).

8. Hassan, A., Macedo, L. J. A., de Souza, J. C. P., Lima, F. C. D. A. & Crespilho, F. N. A combined Far-FTIR, FTIR Spectromicroscopy, and DFT Study of the Effect of DNA Binding on the [4Fe4S] Cluster Site in EndoIII. *Sci. Rep.* **10**, (2020).

9. Pilch-Wrobel, A., Kotulska, A. M., Lahtinen, S., Soukka, T. & Bednarkiewicz, A. Engineering the Compositional Architecture of Core-Shell Upconverting Lanthanide-Doped Nanoparticles for Optimal Luminescent Donor in Resonance Energy Transfer: The Effects of Energy Migration and Storage. *Small* **18**, (2022).

10. Kotulska, A. M., Pilch-Wrobel, A., Lahtinen, S., Soukka, T. & Bednarkiewicz, A. Upconversion FRET quantitation: the role of donor photoexcitation mode and compositional architecture on the decay and intensity based responses. *Light-Sci. Appl.* **11**, (2022).

11. Rong, Y. W. *et al.* An upconversion biosensor based on DNA hybridization and DNA-templated silver nanoclusters for the determination of acrylamide. *Biosens. Bioelectron.* **215**, (2022).

12. Chen, T., Shang, Y. F., Zhu, Y. Y., Hao, S. W. & Yang, C. H. Activators Confined Upconversion Nanoprobe with Near-Unity Forster Resonance Energy Transfer Efficiency for Ultrasensitive Detection. *Acs Appl. Mater. Interfaces* **14**, 19826-19835 (2022).

13. Kang, D., Lee, S., Shin, H., Pyun, J. & Lee, J. An efficient NIR-to-NIR signal-based LRET system for homogeneous competitive immunoassay. *Biosens. Bioelectron.* **150**, (2020).

14. Yan, H., Han, Z., Li, K. M., Li, G. Y. & Wei, X. L. Molecular Dynamics Simulation of the pH-Induced Structural Transitions in CTAB/NaSal Solution. *Langmuir* **34**, 351-358 (2018).

15. Zhou, Y. & Zeng, H. C. Simultaneous Synthesis and Assembly of Noble Metal Nanoclusters with Variable Micellar Templates. *J. Am. Chem. Soc.* **136**, 13805-13817 (2014).

16. Yang, Y. N. *et al.* Multi-shelled Dendritic Mesoporous Organosilica Hollow Spheres: Roles of Composition and Architecture in Cancer Immunotherapy. *Angew. Chem. Int. Edit.* **56**, 8446-8450 (2017).

17. Wang, R. C. *et al.* Precisely Controlled Vertical Alignment in Mesostructured Carbon Thin Films for Efficient Electrochemical Sensing. *Acs Nano* **15**, 7713-7721 (2021).
