# Supplementary material for: Biporous silica nanostructure-induced nanovortex in microfluidics for nucleic acid enrichment, isolation, and PCR-free detection
Source: Nat Commun. 2024 Feb 14;15:1366. doi: 10.1038/s41467-024-45467-w (PMC10866868; doi:10.1038/s41467-024-45467-w)
Supplement: Supplementary file 3 — Description of Additional Supplementary Files [file 41467_2024_45467_MOESM3_ESM.pdf]

DESCRIPTION FOR ADDITIONAL SUPPLEMENTARY FILES DOCUMENT

Supplementary Movie 1. Flow simulation of the PSNF model.

Supplementary Movie 2. Flow simulation of the BSNF model.

Supplementary Movie 3. Flow simulation of the Flat model.

Supplementary Movie 4. Video of the particle proximity test.
