# Supplementary material for: Biporous silica nanostructure-induced nanovortex in microfluidics for nucleic acid enrichment, isolation, and PCR-free detection
Source: Nat Commun. 2024 Feb 14;15:1366. doi: 10.1038/s41467-024-45467-w (PMC10866868; doi:10.1038/s41467-024-45467-w)
Supplement: Supplementary file 8 — Reporting Summary [file 41467_2024_45467_MOESM8_ESM.pdf]

Corresponding author(s): Joonseok Lee
Yong Shin
Rhokyun Kwak

Last updated by author(s): Jan 10, 2024

Reporting Summary

Nature Portfolio wishes to improve the reproducibility of the work that we publish. This form provides structure for consistency and transparency in reporting. For further information on Nature Portfolio policies, see our Editorial Policies and the Editorial Policy Checklist.

Statistics

For all statistical analyses, confirm that the following items are present in the figure legend, table legend, main text, or Methods section.

n/a	Confirmed	
☐	☒	The exact sample size (n) for each experimental group/condition, given as a discrete number and unit of measurement
☐	☒	A statement on whether measurements were taken from distinct samples or whether the same sample was measured repeatedly
☐	☒	The statistical test(s) used AND whether they are one- or two-sided<br>*Only common tests should be described solely by name; describe more complex techniques in the Methods section.*
☒	☐	A description of all covariates tested
☒	☐	A description of any assumptions or corrections, such as tests of normality and adjustment for multiple comparisons
☐	☒	A full description of the statistical parameters including central tendency (e.g. means) or other basic estimates (e.g. regression coefficient) AND variation (e.g. standard deviation) or associated estimates of uncertainty (e.g. confidence intervals)
☐	☒	For null hypothesis testing, the test statistic (e.g. F, t, r) with confidence intervals, effect sizes, degrees of freedom and P value noted<br>*Give P values as exact values whenever suitable.*
☒	☐	For Bayesian analysis, information on the choice of priors and Markov chain Monte Carlo settings
☒	☐	For hierarchical and complex designs, identification of the appropriate level for tests and full reporting of outcomes
☒	☐	Estimates of effect sizes (e.g. Cohen's d, Pearson's r), indicating how they were calculated

Our web collection on statistics for biologists contains articles on many of the points above.

Software and code

Policy information about availability of computer code

Data collection	SEM images were obtained using a ZEISS Sigma 300 field-emission SEM (FE-SEM) (ZEISS, Germany). The hydrodynamic diameter and zeta potential was measured by a Zetasizer Nano ZSP (Malvern, UK). SAXS measurement was performed using a Xeuss 2.0 (Xenocs, France). Fluorescence images were observed under fluorescence imaging system (EVOS M7000 Imaging System, Thermo Fisher Scientific, USA). FT-IR spectrum was obtained using a PerkinElmer Frontier™ FT-IR spectrometer with a diamond ATR (Perkin Elmer, USA). The water contact angle was measured using pendant drop tensiometer DSA100 (Kruss, Germany). The trajectories of the particles were captured using an inverted microscopy (IX-71, Olympus Co., Japan) and EMCCD (ImagEMx2, Hamamatsu Co., Japan). The amplified products were detected through SYBR green and Cy5 fluorescence signals using the CFX96 Real-Time PCR System (Bio-Rad, Hercules, CA, USA). The morphology of the LnNPs was analyzed on a JEM-2100F (JEOL Ltd., Japan). The XRD patterns of the LnNPs were characterized by an XRD-7000 diffractometer. The Fourier transform infrared (FT-IR) spectra of the LnNPs were obtained by using a Nicolet iS50 FT-IR spectrophotometer (Thermo Fisher Scientific Co., USA). The photoluminescence (PL) emission spectra were recorded by a spectrometer (Andor, Kymera 193i) and an intensified sCMOS detector (Andor, ISTAR-SCMOS-18F-73). The PL lifetime was measured using a photomultiplier tube detector (H10721-01; Hamamatsu, Shizuoka, Japan) attached to the spectrometer and a digital oscilloscope (Rhode & Schwarz, Munich, Germany, RTM3002).
Data analysis	Computational modeling and simulations were carried out via the commercial solver of the GeoDict® 2023 software package (Math2Market GmbH, Germany). Feret diameter and mean gray value were analyzed in ImageJ software. Statistical analyses were performed using Origin Pro 2016 and IBM SPSS statistical package (version 27).

For manuscripts utilizing custom algorithms or software that are central to the research but not yet described in published literature, software must be made available to editors and reviewers. We strongly encourage code deposition in a community repository (e.g. GitHub). See the Nature Portfolio guidelines for submitting code & software for further information.

Data

Policy information about availability of data

All manuscripts must include a data availability statement. This statement should provide the following information, where applicable:

- Accession codes, unique identifiers, or web links for publicly available datasets
- A description of any restrictions on data availability
- For clinical datasets or third party data, please ensure that the statement adheres to our policy

All data that support the findings of this study are available within the Article, Supplementary Information or Source Data File. Source data are provided with this paper.

Research involving human participants, their data, or biological material

Policy information about studies with human participants or human data. See also policy information about sex, gender (identity/presentation), and sexual orientation and race, ethnicity and racism.

Reporting on sex and gender	Sex or gender were not considered as influencing factors in the design aimed at improving detection sensitivity.
Reporting on race, ethnicity, or other socially relevant groupings	Differences based on these characteristics were not analyzed.
Population characteristics	COVID-19 Positive (n = 20): Median Age = 61 [IQR 48-66], 45% male; COVID-19 Negative (n = 10): Median Age = 60 [IQR 45-64], 50% male.
Recruitment	The clinical specimens were collected from Asan Medical Center (Seoul, Korea) between March and November 2022. We meticulously collected clinical specimens from participants who were confirmed to have SARS-CoV-2 infection via nasopharyngeal RT-PCR. Our protocol involved conducting weekly RT-PCR tests on a variety of respiratory samples, which included nasopharyngeal swabs, saliva, or sputum. This routine was maintained for a duration of up to 12 weeks. In cases where RT-PCR results continued to show positive outcomes after the 12-week mark, we extended the testing on a weekly basis until the participants produced two consecutive negative results.
Ethics oversight	The study protocol was reviewed and approved by the Institutional Review Board of Asan Medical Center, Seoul, Korea (IRB-2022-1054).

Note that full information on the approval of the study protocol must also be provided in the manuscript.

Field-specific reporting

Please select the one below that is the best fit for your research. If you are not sure, read the appropriate sections before making your selection.

☒ Life sciences ☐ Behavioural & social sciences ☐ Ecological, evolutionary & environmental sciences

For a reference copy of the document with all sections, see nature.com/documents/nr-reporting-summary-flat.pdf

Life sciences study design

All studies must disclose on these points even when the disclosure is negative.

Sample size	We randomly collected 30 nasopharyngeal swabs in this study, including 20 positive cases and 10 negative cases. Sample sizes (20-40 samples for human samples) were chosen based on what is common practice in the field. Statistic provided figure of sample size was performed at least 3-times independent experiments. And also, sample size was stated in the all figure captions.
Data exclusions	No data were excluded from the analyses.
Replication	At least three replicates were analyzed in each independent experiment to ensure the experimental results were reliable. Biological and technical replicates were considered. At least 2 or 3 independent experiments were performed to validate key data.
Randomization	To ensure unbiased results, samples are randomly allocated.
Blinding	Blinding was not employed in this study as we did not consider it necessary to mitigate potential subjectivity of the researcher. We applied the same measurements and analysis techniques uniformly to all experimental groups, which minimized the potential for bias

Reporting for specific materials, systems and methods

We require information from authors about some types of materials, experimental systems and methods used in many studies. Here, indicate whether each material, system or method listed is relevant to your study. If you are not sure if a list item applies to your research, read the appropriate section before selecting a response.

Materials & experimental systems

n/a	Involved in the study
☒	☐ Antibodies
☐	☒ Eukaryotic cell lines
☒	☐ Palaeontology and archaeology
☒	☐ Animals and other organisms
☒	☐ Clinical data
☒	☐ Dual use research of concern
☒	☐ Plants

Methods

n/a	Involved in the study
☒	☐ ChIP-seq
☒	☐ Flow cytometry
☒	☐ MRI-based neuroimaging

Eukaryotic cell lines

Policy information about cell lines and Sex and Gender in Research

Cell line source(s)	Human HCT116 (Sex:male, KCLB No.10247) was purchased from Korean Cell Line Bank (Seoul, Korea).
Authentication	None of the cell lines was authenticated.
Mycoplasma contamination	The cell lines were regularly tested and confirmed to be free from mycoplasma contamination.
Commonly misidentified lines (See ICLAC register)	Non of the misidentified lines were used in this study.

Plants

Seed stocks	*Report on the source of all seed stocks or other plant material used. If applicable, state the seed stock centre and catalogue number. If plant specimens were collected from the field, describe the collection location, date and sampling procedures.*
Novel plant genotypes	*Describe the methods by which all novel plant genotypes were produced. This includes those generated by transgenic approaches, gene editing, chemical/radiation-based mutagenesis and hybridization. For transgenic lines, describe the transformation method, the number of independent lines analyzed and the generation upon which experiments were performed. For gene-edited lines, describe the editor used, the endogenous sequence targeted for editing, the targeting guide RNA sequence (if applicable) and how the editor was applied.*
Authentication	*Describe any authentication procedures for each seed stock used or novel genotype generated. Describe any experiments used to assess the effect of a mutation and, where applicable, how potential secondary effects (e.g. second site T-DNA insertions, mosiacism, off-target gene editing) were examined.*
